# Epigenome-wide Analysis Identifies Genes and Pathways Linked to Neurobehavioral Variation in Preterm Infants

**DOI:** 10.1038/s41598-019-42654-4

**Published:** 2019-04-19

**Authors:** Todd M. Everson, Carmen J. Marsit, T. Michael O’Shea, Amber Burt, Karen Hermetz, Brian S. Carter, Jennifer Helderman, Julie A. Hofheimer, Elisabeth C. McGowan, Charles R. Neal, Steven L. Pastyrnak, Lynne M. Smith, Antoine Soliman, Sheri A. DellaGrotta, Lynne M. Dansereau, James F. Padbury, Barry M. Lester

**Affiliations:** 1 Department of Environmental Health, Emory University Rollins School of Public Health, Atlanta, GA United States; 2 Department of Pediatrics, University of North Carolina School of Medicine, Chapel Hill, NC United States; 3 Department of Pediatrics-Neonatology, Children’s Mercy Hospital, Kansas City, MO United States; 4 Department of Pediatrics, Wake Forest School of Medicine, Winston Salem, NC United States; 5 Department of Pediatrics, Brown Alpert Medical School and Women and Infants Hospital, Providence, RI United States; 6 Department of Pediatrics, University of Hawaii John A. Burns School of Medicine, Honolulu, HI United States; 7 Department of Pediatrics, Spectrum Health-Helen Devos Hospital, Grand Rapids, MI United States; 8 Department of Pediatrics, Harbor-UCLA Medical Center, Torrance, CA United States; 9 Department of Pediatrics, Miller Children’s and Women’s Hospital Long Beach, Long Beach, CA United States; 10 Brown Center for the Study of Children at Risk, Brown Alpert Medical School and Women and Infants Hospital, Providence, RI United States; 11 Department of Psychiatry and Human Behavior, Brown Alpert Medical School, Providence, RI United States

**Keywords:** Neurodevelopmental disorders, DNA methylation

## Abstract

Neonatal molecular biomarkers of neurobehavioral responses (measures of brain-behavior relationships), when combined with neurobehavioral performance measures, could lead to better predictions of long-term developmental outcomes. To this end, we examined whether variability in buccal cell DNA methylation (DNAm) associated with neurobehavioral profiles in a cohort of infants born less than 30 weeks postmenstrual age (PMA) and participating in the Neonatal Neurobehavior and Outcomes in Very Preterm Infants (NOVI) Study (N = 536). We tested whether epigenetic age, age acceleration, or DNAm levels at individual loci differed between infants based on their NICU Network Neurobehavioral Scale (NNNS) profile classifications. We adjusted for recruitment site, infant sex, PMA, and tissue heterogeneity. Infants with an optimally well-regulated NNNS profile had older epigenetic age compared to other NOVI infants (β_1_ = 0.201, p-value = 0.026), but no significant difference in age acceleration. In contrast, infants with an atypical NNNS profile had differential methylation at 29 CpG sites (FDR < 10%). Some of the genes annotated to these CpGs included *PLA2G4E*, *TRIM9*, *GRIK3*, and *MACROD2*, which have previously been associated with neurological structure and function, or with neurobehavioral disorders. These findings contribute to the existing evidence that neonatal epigenetic variations may be informative for infant neurobehavior.

## Introduction

Preterm birth is a significant global public health problem. In the United States one in eight children are born less than 37 weeks of gestation^1^. Survival of infants born very preterm, prior to 30 weeks postmenstrual age (PMA), has improved due to technological and medical advancements^2,3^. These youngest infants are more likely to suffer from chronic illnesses, potentially devastating brain injuries, and adverse neuromotor, cognitive, and behavioral outcomes that persist through adulthood^4–15^. These consequences of premature birth often require extensive healthcare, educational and psychosocial community resources, in addition to increased burden on the families and caregivers of these children, emotionally and financially.

In addition to immaturity, premature infants vary widely in the health complications they experience. As such, assessments prior to discharge from the neonatal intensive care unit (NICU) are needed to identify the earliest risks for adverse neurodevelopmental outcomes, and to maximize the potential benefits of interventions aimed at ameliorating long term deficits. There is growing evidence that neonatal neurobehavior (the relationships between the nervous system and behavior), as measured by the NICU Network Neurobehavioral Scale (NNNS)^16^, predicts developmental deficits in infants born preterm and others at risk, beyond what can be predicted based on the assessment of medical risk factors throughout the newborn’s hospital stay^17–20^. Latent profiles of NNNS summary scores have been used to classify infants into groups with similar responses across the overall NNNS assessment. These neurobehavioral profiles in infants have been associated with prenatal exposures (prenatal drugs^21,22^ and perfluorooctanoic acid^23^), birth outcomes (gestational age and birth weight^21^), and with behavioral and cognitive outcomes in childhood^21,22,24^. Poorer performance on the NNNS has also been shown to be predictive of non-optimal developmental outcomes through early childhood^21^. Beyond the neurobehavioral and medical assessments, molecular biomarkers may provide insights into how the environment and experiences of the preterm newborn are internalized and may hold additional value as predictive tools useful in risk stratification.

Epigenetics refers to mitotically and meiotically heritable changes in gene expression potential that are not explained by changes in DNA sequence. The most thoroughly studied epigenetic mechanism is DNA methylation (DNAm), particularly in the context of cytosine-phosphate-guanine (CpG) motifs and islands. These methylation marks can be inherited across cell divisions, established in-utero and/or affected by the environment throughout life, thus representing a truly integrated measure of exposure and disease susceptibility. In preterm infants, variability in DNAm of candidate genes have been related to medical complications such as sepsis^25^, pain related stress^26,27^, medical and neurobehavioral risk^28,29^, and as a potential moderator of NICU environment stress on serotonergic tone and temperament^30^. We have also used an epigenome-wide scan of DNAm in the placenta to demonstrate relationships between methylation of the *FHIT* and *ANDKR11* genes, which had been previously linked to neurodevelopmental and behavioral outcomes, and performance on the NNNS attention scale in a cohort of term newborns^31^. Additionally, DNAm can be used to estimate epigenetic age, which is thought to be a marker of underlying biological aging^32^. In children and adolescents, epigenetic age acceleration has been associated with physical development^33^, pubertal development, internalization and thought problems^34^, and increased cortisol production^34,35^. These studies have begun to elucidate the potential role for epigenetic aging in developmental processes but has not been thoroughly studied in relation to neonatal neurobehavioral responses.

Incorporating molecular biomarkers, such as DNAm and epigenetic age, with performance measures may improve long-term predictions of health outcomes in preterm infants. Before this can be done, it is important to demonstrate whether variability in DNAm and epigenetic age, measured from accessible tissues, are associated with behavioral measures in newborns. In this study, we hypothesized that infants with the most atypical or optimal neurobehavioral profiles, measured via the NNNS, will exhibit unique patterns of DNAm. In a U.S. multisite cohort of infants born less than 30 weeks PMA, we profiled genome-wide DNAm from buccal swab samples using the Illumina MethylationEPIC array platform. We estimated epigenetic age and tested for differences in epigenetic age and in DNAm among infants with neurobehavioral profiles, ranging from most optimal to atypical, as determined via NNNS latent profile classification.

## Methods

### Study Population

The Neonatal Neurobehavior and Outcomes in Very Preterm Infants (NOVI) Study was conducted at 9 university-affiliated NICUs in Providence, RI, Grand Rapids, MI, Kansas City, MO, Honolulu, HI, Winston-Salem, NC, and Torrance and Long Beach CA from April 2014 through June 2016. These NICUs were also Vermont Oxford Network (VON) participants. All participating mothers provided written informed consent. Enrollment and consent procedures for this study were approved by the institutional review boards of Women and Infants Hospital, Spectrum Health, Children’s Mercy Office of Research Integrity, Wake Forest University Health Sciences, John F. Wolf, MD Human Subjects Committee at Los Angeles BioMed, Emory University and Western Institutional Review Board (WIRB); all methods employed in the study were performed in accordance with the relevant guidelines and regulations. Eligibility was determined based on the following inclusion criteria: (1) birth at <30 weeks’ gestation; (2) parental ability to read and speak English, Spanish, Japanese, or Chinese; and (3) residence within 3 hours of the NICU and follow-up clinic. Infants were excluded if their medical record indicated presence of a major congenital anomaly, including central nervous system, cardiovascular, gastrointestinal, genitourinary, chromosomal, and nonspecific anomalies^36^. Parents of eligible infants were invited to participate in the study at 31–32 weeks PMA, or when survival to discharge was determined to be likely by the attending neonatologist. Demographic variables, including infant gender, race, ethnicity, maternal education and partner status were collected from the maternal interview. Socioeconomic status (SES) was assessed using the Hollingshead Index, with Hollingshead level V indicating low SES^37^. Neonatal medical variables including birthweight, gestational age, length of NICU stay, weight at discharge, and gestational age at discharge were abstracted from medical records. Overall, 709 infants were enrolled, 679 from whom complete neurobehavioral assessment data were obtained, and buccal cells were collected on 624 of these infants for epigenomic screening.

### NICU Network Neurobehavioral Scale (NNNS)

Neonatal neurobehavior was assessed using the NNNS. The NNNS is a 20–30 minute standardized procedure that includes measures of active and passive tone, primitive reflexes, items that reflect physical maturity, social and behavioral functioning including visual and auditory tracking, cuddling and soothability, and a checklist of stress signs organized by organ system^38^. The NNNS was administered during the week of NICU discharge (+/− 3 days) by site examiners who were trained and certified by a central NOVI NNNS trainer. The exam was conducted 45 minutes prior to a scheduled feeding or routine care in order to maximize alertness and avoid disrupting NICU routines that facilitate sleep patterns. Individual items were converted to 12 summary scores: attention, handling, self-regulation, arousal, excitability, lethargy, hypertonicity, hypotonicity, non-optimal reflexes, asymmetric reflexes, quality of movement and stress abstinence. A higher summary score does not necessarily reflect better performance, but instead more of the construct. Summary scores were converted to NNNS profiles, which are mutually exclusive, discrete categories representing the infant’s pattern of performance across the summary scores, and which have been shown to be related to future behavioral and cognitive performance^21,22,24^.

### DNA Methylation (DNAm) Analysis

Genomic DNA was extracted from buccal swab samples, collected near term-equivalent age, using the Isohelix Buccal Swab system (Boca Scientific), quantified using the Quibit Fluorometer (Thermo Fisher, Waltham, MA, USA) and aliquoted into a standardized concentration for subsequent analyses. DNA samples were plated randomly across 96-well plates and provided to the Emory University Integrated Genomics Core for bisulfite modification using the EZ DNA Methylation Kit (Zymo Research, Irvine, CA), and subsequent assessment of genome-wide DNAm using the Illumina MethylationEPIC Beadarray (Illumina, San Diego, CA) following standardized methods based on the manufacturer’s protocol. Samples with more than 5% of probes yielding detection p-values > 1.0E-5 (74 samples), with mismatch between reported and predicted sex (7 samples), or incomplete covariate data (7 samples) were excluded. Additionally, probes with median detection p-values < 0.05 were excluded. Array data were normalized via functional normalization, then standardized across Type-I and Type-II probe designs with beta-mixture quantile normalization^39^. Probes that measured methylation on the X and Y chromosomes, probes that had single nucleotide polymorphisms (SNP) within the binding region, that could cross-hybridize to other regions of the genome^40^, or probes that had low variability (range of beta-values < 0.05)^41^ were excluded. After exclusions, 690,781 probes were available from 536 samples for this study. We used gaphunter to flag probes that had outliers or distributional issues that may be related to genetic effects on DNAm measurement^42^. These data are accessible through NCBI Gene Expression Omnibus (GEO) via accession series GSE128821.

### Estimate of Epigenetic Age

We estimated epigenetic age using the online (https://horvath.genetics.ucla.edu/html/dnamage/) [Accessed 01/15/2019] epigenetic clock calculator^43^. This method utilizes DNAm levels at previously identified CpGs that are predictive of chronological age and has been shown to be highly accurate across a wide range of different cell and tissue types^32^. This clock also calculates two measures of age acceleration: the difference between epigenetic and chronological age, and the residuals when epigenetic age is regressed on chronological age in a linear model. We investigated the age acceleration residuals and epigenetic age in this study.

### Estimates of Tissue Heterogeneity

DNAm differs between cell-types, and cellular heterogeneity presents a likely source of confounding in epigenome-wide association studies of mixed cell samples^44^. Thus, we estimated the proportions of epithelial, fibroblast, and immune cells (including B-cells, natural killers, CD4+ T-cells, CD8+ T-cells, monocytes, neutrophils, and eosinophils) in our cheek swab samples using reference methylomes^45^. For 95% of our samples, epithelial cells made up 95.7% of the cells (Supplemental Fig. 1), while immune cells made up the majority of the remaining cell types. Due to very strong inverse correlations between immune cell proportions and epithelial cell proportions (Supplemental Fig. 2), we adjusted for cellular heterogeneity by including the proportions of epithelial cells as covariates in the statistical models.

### Statistical Analyses

Latent profile analysis (LPA, Mplus version 8.1) was used to group infants into mutually exclusive categories using 12 NNNS summary scores based on previous work^21^. Membership in categorical latent profiles that represent heterogeneous subgroups was inferred from the 12 NNNS variables. LPA models with different numbers of profiles were fitted. We identified the model containing the optimal number of profiles using criteria outlined by Nylund *et al*.^46^. Determination of the best model fit was assessed via Bayesian information criteria (BIC) adjusted for sample size, whereby the smallest BIC value indicates the best fit as well as minimization of cross classification probabilities, the bootstrapped likelihood ratio test, and the number of cases in each profile. As the number of profiles increased from 2 to 6, the sample-size adjusted BIC values decreased, suggesting improvement in the goodness of fit (Supplemental Table 1). Infants in Profile 6 had a pattern of responses (lowest scores for attention, self-regulation and quality of movement, average scores for lethargy, hypotonicity, nonoptimal reflexes and asymmetric reflexes, combined with the highest scores for arousal, excitability, hypertonicity and the most stress abstinence) that was consistent with a latent profile identified by Liu *et al*. (2010) that had the most extreme negative scores and was most predictive of nonoptimal developmental outcomes in childhood^21^. Whereas infants in Profile 1 exhibited the most optimal responses, and provided the greatest contrast to Profile 6, thus we focused on these two profiles for the EWAS and epigenetic age analyses.

Statistical analyses of epigenomic data were performed in R version 3.5. We tested for differences in epigenetic age and age acceleration between the atypical NNNS profile (Profile 6) and optimal NNNS profile (Profile 1) versus those in the other NNNS profiles using robust linear models via the MASS package. Standard errors and p-values for robust regressions were estimated using White’s sandwich estimator to protect against potential heteroscedasticity. Epigenetic age and age acceleration were included as continuous dependent variables, the NNNS profiles were included as a three-level factor for the independent variable, while adjusting for sex, recruitment site, and cellular heterogeneity. The epigenome-wide association study was performed with robust linear regressions for each CpG site that passed QC, regressing methylation beta-values (dependent variable) on the NNNS profiles (independent variable), while adjusting for sex, PMA, and proportions of epithelial cells and fibroblasts. QQ-plots and Manhattan plots were produced using the qqman package. To account for multiple testing, we implemented a false discovery rate (FDR) and considered those associations that were within a 10% FDR to be statistically significant. We report all results from models that yielded suggestive associations (p-value < 0.0001) in the Supplemental Materials.

We performed sensitivity analyses to examine the impacts of other potential confounders on the associations between NNNS profiles and DNAm. For these analyses, we included additional variables in the linear models, and compared the parameter estimates before and after these additional adjustments. We assessed the confounding effects of sample plate as a batch variable (7-level factor), maternal socioeconomic status (2-level factor), maternal educational attainment (2-level factor), proportions of fibroblasts and immune cells (B-cells, natural killers, CD4+ T-cells, CD8+ T-cells, monocytes, neutrophils, and eosinophils), race (white, black, Asian, Hawaiian/Pacific Islander, and other), and birth weight (grams).

To gain insights into the biological functions of the NNNS-associated CpG sites, we performed over-representation analyses with ConsensusPathDB (CPDB)^47,48^. We utilized CPDB to examine our gene lists for enrichment with neighborhood-based entity sets (NESTs) with a radius of one, pathway-based gene sets from KEGG, Biocarta, and Reactome with minimum overlap with our gene-set of 2, and gene-ontology (GO) terms. Over-representation results within a 10% FDR were determined to be statistically significant. For over-representation analyses, we utilized a gene-list containing the genes annotated to the top 250 CpG sites from the EWAS that were associated with the atypical NNNS profile. We also aimed to examine whether our NNNS-associated CpGs were within genes that have been linked with phenotypes related to neurodevelopment or neurodegeneration. Thus, we annotated the top 250 CpGs with traits that have been linked to genes via the NHGRI-EBI genome-wide association study catalog (GWAS catalog)^49^.

## Results

### Study Population and NNNS Profile Results

**Figure 1:**
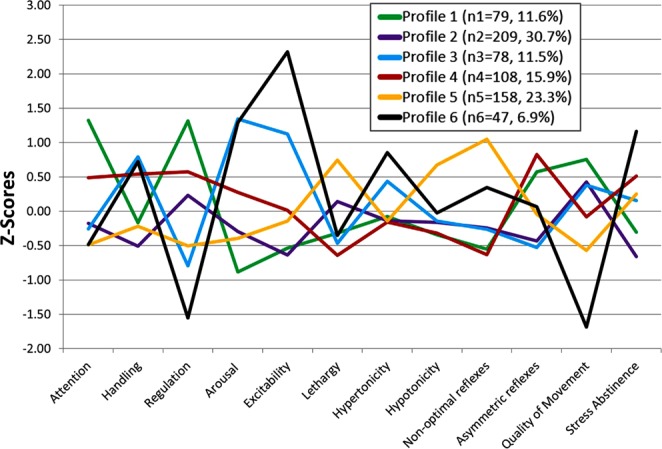
Patterns of NNNS z-scores across individual assessments for all six latent profiles among all NOVI infants that were assessed for the NNNS (N = 679); Profile 6 represents the atypical profile (black) and Profile 1 represents the optimal profile (green).

We identified six distinct NNNS profiles representing groups of infants with similar neurobehavioral responses (Fig. 1 and Table 1); two of these profiles stood out as particularly distinctive. Infants in Profile 1 had the most optimal performance, with the best attention and regulations scores, an average requirement for handling, typical motor tone and movement, and few signs of stress. Infants in Profile 6 showed atypical performance with poor attention, a substantial requirement for handling, poor regulation, exceptionally high arousal and excitability, hypertonia, poor quality of movement, and substantial signs of stress. Thus, Profile 1 represents an optimal profile characterized by generally positive, well-modulated neurobehavioral responses, while Profile 6 represents infants with atypical neurobehavioral responses. These findings are similar to profiles observed previously by others^21^ and in our own research^29^. To limit the number of tests being performed, the current study focused on the most optimal (Profile 1) and atypical (Profile 6) profiles, while using the combination of Profiles 2–5 as the referent category in downstream analyses. Average PMA at birth, PMA at buccal cell collection, and maternal age did not substantially differ between the different NNNS profile groupings (Table 2). On the other hand, we did find that a larger proportion of infants with atypical profiles had caregivers with lower socioeconomic status (SES) (16.7%) and lower educational attainment (22.2%), compared to those in the optimal group (4.8% and 6.5% respectively). We also observed significant differences in NNNS profile assignment by recruitment site, and thus recruitment site was controlled for in all downstream analyses.

**Table 1:** Means and standard errors of individual NNNS assessment scores across the NNNS profile groupings identified by LPA (N = 536).

Assessment	Profiles						ANOVA p-val.
	1 (n = 62)	2 (n = 176)	3 (n = 61)	4 (n = 83)	5 (n = 118)	6 (n = 36)	
Attention	7.27 (1.14)	5.04 (1.22)	4.89 (0.90)	6.04 (1.18)	4.43 (1.36)	4.36 (1.25)	<0.001
Handling	0.37 (0.25)	0.29 (0.22)	0.61 (0.25)	0.55 (0.22)	0.37 (0.26)	0.62 (0.27)	<0.001
Quality of Movement	5.06 (0.55)	4.89 (0.45)	4.9 (0.59)	4.58 (0.58)	4.23 (0.49)	3.4 (0.64)	<0.001
Self Regulation	6.69 (0.60)	5.82 (0.45)	5.04 (0.53)	6.09 (0.58)	5.21 (0.51)	4.44 (0.72)	<0.001
Non-Optimal Reflexes	4.18 (1.61)	4.83 (1.52)	4.84 (1.67)	4.1 (1.50)	7.47 (1.70)	6.22 (1.93)	<0.001
Stress Abstinence	0.11 (0.06)	0.09 (0.06)	0.14 (0.07)	0.17 (0.06)	0.16 (0.06)	0.22 (0.08)	<0.001
Arousal	3.18 (0.48)	3.55 (0.37)	4.59 (0.51)	3.91 (0.50)	3.47 (0.50)	4.61 (0.51)	<0.001
Hypertonicity	0.34 (0.75)	0.27 (0.55)	0.8 (1.15)	0.25 (0.49)	0.23 (0.53)	0.97 (1.18)	<0.001
Hypotonicity	0.05 (0.22)	0.15 (0.36)	0.13 (0.39)	0.06 (0.29)	0.52 (0.72)	0.25 (0.44)	<0.001
Asymmetrical Reflexes	1.69 (1.52)	0.43 (0.73)	0.25 (0.54)	2.05 (1.51)	0.94 (1.15)	0.97 (1.44)	<0.001
Excitability	1.42 (1.15)	1.16 (0.93)	4.77 (1.27)	2.52 (1.35)	2.18 (1.13)	7.22 (1.61)	<0.001
Lethargy	3.77 (1.58)	4.8 (1.87)	3.64 (1.53)	3.17 (1.58)	6.17 (2.15)	3.97 (1.92)	<0.001

ANOVA: analysis of variance, LPA: latent profile analysis, NNNS: NICU network neurobehavioral scale.

**Table 2:** Means ± standard deviations (continuous) or percentages and frequencies (categorical) of covariates by NNNS profile groupings.

Sample Characteristics	Profiles 2,3,4,5 (N = 438)	Profile 1 (Optimal) (N = 62)	Profile 6 (Atypical) (N = 36)
Infant Gender: Male	55.3% (242/438)	56.5% (35/62)	61.1% (22/36)
Recruitment Site:			
WIH	19.4% (85/438)	3.2% (2/62)	19.4% (7/36)
SHD	22.6% (99/438)	1.6% (1/62)	0.0% (0/36)
KMC	16.0% (70/438)	17.7% (11/62)	19.4% (7/36)
CMH*	14.8% (65/438)	8.1% (5/62)	0.0% (0/36)
WFU*	14.6% (64/438)	67.7% (42/62)	22.2% (8/36)
LAB*	12.6% (55/438)	1.6% (1/62)	38.9% (14/36)
PMA at Buccal Collection (weeks)	38.930 ± 3.150	39.415 ± 4.012	40.210 ± 3.462
PMA at Birth (weeks)	27.091 ± 1.881	26.756 ± 1.954	26.452 ± 2.154
Birth Weight (grams)	969.0 ± 281.4	903.8 ± 265.0	861.9 ± 291.4
Maternal Age at childbirth (years)	29.171 ± 6.297	29.095 ± 6.972	27.972 ± 6.396
Maternal Smoking During Pregnancy	16.3% (71/436)	14.8% (9/61)	8.3% (3/36)
Education: less than High School/GED	14.6% (62/426)	6.5% (4/62)	22.2% (8/36)
Low SES	8.2% (35/427)	4.8% (3/62)	16.7% (6/36)
Maternal Race:			
White	55.0% (236/429)	52.5% (32/61)	27.8% (10/36)
Black	21.9% (94/429)	29.5% (18/61)	19.4% (7/36)
Asian	7.0% (30/429)	9.8% (6/61)	11.1% (4/36)
Hawaiian or Pacific Islander	6.8% (29/429)	6.6% (4/61)	13.9% (5/36)
Other	9.3% (40/429)	1.6% (1/61)	27.8% (10/36)

PMA: Postmenstrual Age, SES: socioeconomic status, GED: General Equivalency Diploma, NNNS: NICU network neurobehavioral scale, *enrolled from 2 university-affiliated NICUs.

### Epigenetic Age and Age Acceleration Associations with NNNS Profiles

**Figure 2:**
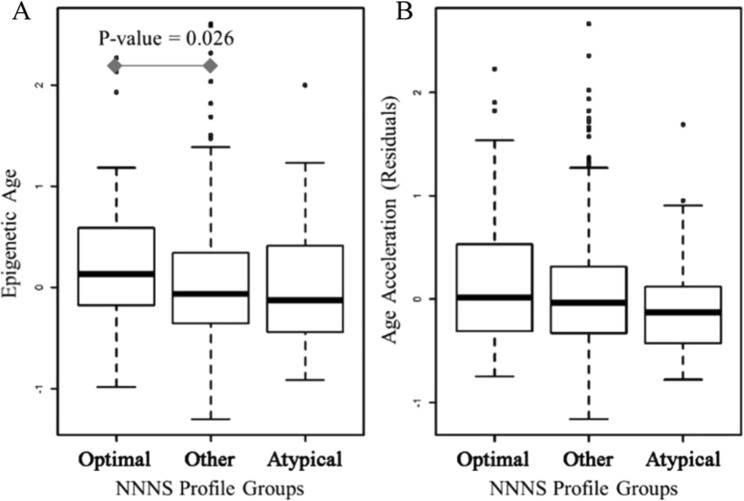
Relationships between epigenetic age (**A**) and age acceleration (**B**) with NNNS profile groupings; the x-axis includes the optimal profile (Profile 1), the atypical profile (Profile 6), as well as other NOVI infants (Profiles 2–5), while the y-axis represents epigenetic age and age acceleration after adjusting for sex, recruitment site, and cell-mixture.

Epigenetic age negatively correlated with gestational age at birth (R^2^ = 0.06, p-value < 0.0001), but positively correlated with age since birth (R^2^ = 0.15, p-value < 0.0001) and PMA (R^2^ = 0.14, p-value < 0.0001) (Supplemental Fig. 3). In a linear model with epigenetic age regressed on both gestational age at birth and PMA at exam date, the relationship with PMA remained strong while the associations with gestational age was heavily attenuated. Thus, PMA appeared to be the appropriate age variable to include in models when testing for age acceleration. We examined differences in epigenetic age and age acceleration that associated with the optimal (n = 62) and atypical (n = 36) NNNS profiles, by comparing them to the rest of the NOVI infants (n = 438). We found that the infants in the optimal profile tended to have significantly older epigenetic age (β_1_ = 0.201, p-value = 0.026) whereas the atypical profile exhibited no difference in epigenetic age (β_1_ = −0.022, p-value = 0.84) when compared to the rest of the NOVI infants (Fig. 2A). However, age acceleration did not significantly differ when comparing the optimal or atypical profiles to the rest of the NOVI infants (Fig. 2B). These models were adjusted for sex, recruitment site, postmenstrual age, and estimated proportions of epithelial cells and fibroblast cells.

### Epigenome-Wide Association Study of NNNS Profiles

**Figure 3:**
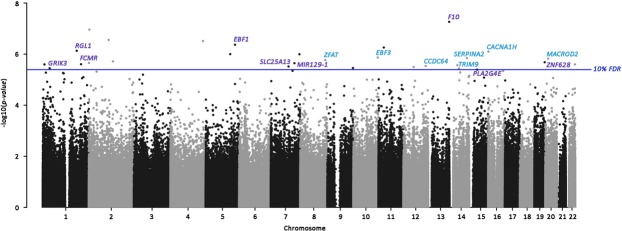
Manhattan plot of epigenetic loci associated with the atypical NNNS profile; the x-axis represents the genomic location of the individual probes and the y-axis represents the -log_10_(p-values) from related to the Atypical NNNS profile, adjusted for sex, recruitment site, postmenstrual age, and cell-mixture; gene annotations for the CpGs yielding associations within the 10% FDR threshold have been added to the plot.

We then performed an EWAS of these two NNNS profiles to examine whether underlying patterns of epigenetic regulation measured in buccal cells differed among infants within the NNNS profiles. We report all results for the EWAS of the optimal and atypical NNNS profiles that yielded associations with p-values < 0.0001 in the Supplemental Materials (Supplemental Tables 2, 3, respectively). Thirty CpGs were differentially methylated (at a 10% FDR) with either of the NNNS profile groupings (Table 3). The only CpG associated with the optimal NNNS profile was cg03046148. Infants with this profile had higher DNAm levels (β_1_ = 0.0145, p-value = 1.43E-07). On the other hand, we identified 29 epigenetic loci that were associated with the atypical NNNS profile after FDR-adjustment, which were located throughout the genome (Fig. 3). The most statistically significant relationship was observed at cg23172057 (β_1_ = 0.0299, p-value = 5.43E-08) which is within the body of the coagulation factor X (*F10*) gene. The magnitudes of effect among the FDR-significant hits tended to be small, with differential methylation ranging between 0.42% to 6.53% lower and between 0.31% to 10.61% higher methylation among the atypical NNNS group. The CpGs with the largest magnitudes of association among the FDR-significant hits were observed at cg14792155 (β_1_ = 0.1061, p-value = 4.04E-06) which is within the body of the phospholipase A2 group IVE (*PLA2G4E*) gene and at cg07850633 (β_1_ = −0.0653, p-value = 1.49E-06) which is within the body of the MACRO domain containing 2 (*MACROD2*) gene.

**Table 3:** Epigenome-wide association study results for CpG sites that yielded associations within a 5% (**) or 10% (*) FDR for either the optimal or atypical NNNS profile groupings; beta coefficients (β_1_) represent the mean difference in methylation proportion when comparing the optimal or atypical NNNS profiles to the rest of the NOVI sample.

CpG Annotations			Optimal NNNS Profile		Atypical NNNS Profile	
CpG	Location	Gene	β_1_	P-value	β_1_	P-value
cg25755851	chr1:9335794	*—*	−0.013	1.89E-01	−0.032	2.53E-06*
cg22960767	chr1:37422679	*GRIK3 (Body)*	0.022	1.39E-02	0.036	3.55E-06*
cg26057754	chr1:183774231	*RGL1 (Body)*	−0.001	7.00E-01	−0.004	7.38E-07*
cg23264395	chr1:207096239	*FCMR (TSS1500)*	0.001	8.50E-01	0.034	2.48E-06*
cg01479768	chr2:731298	*—*	−0.006	6.91E-01	0.045	2.20E-06*
cg08902894	chr2:3142407	*—*	0.002	7.22E-01	0.029	1.10E-07*
cg05696361	chr2:107108978	*—*	0.003	7.78E-01	0.048	2.79E-07*
cg17505883	chr2:130552292	*—*	0.006	2.59E-01	0.021	1.94E-06*
cg03046148	chr3:13695666	*LOC285375 (Body)*	0.015	1.43E-07*	0.003	4.56E-01
cg07193729	chr4:176031198	*—*	0.006	5.03E-02	0.014	3.07E-07*
cg02236672	chr5:132449216	*—*	−0.001	9.31E-01	0.037	9.98E-07*
cg00210856	chr5:158466226	*EBF1 (Body)*	0.001	9.23E-01	0.028	4.29E-07*
cg02057469	chr7:95951623	*SLC25A13 (TSS200)*	−0.001	1.19E-01	−0.004	3.05E-06*
cg04524088	chr7:127847835	*MIR129-1 (TSS200)*	−0.016	1.68E-01	0.045	2.27E-06*
cg17287134	chr7:154959606	*—*	−0.008	4.52E-01	0.040	1.01E-06*
cg21672855	chr8:135614777	*ZFAT (Body)*	−0.002	1.73E-01	0.005	1.71E-06*
cg14632902	chr9:139017648	*—*	0.012	1.91E-02	0.026	3.49E-06*
cg06846137	chr10:131682939	*EBF3 (Body)*	0.004	4.20E-01	0.019	1.36E-06*
cg13716458	chr11:28997975	*—*	0.006	6.38E-02	0.020	5.51E-07*
cg07895260	chr12:55537168	*—*	−0.001	9.37E-01	0.034	3.19E-06*
cg27361636	chr12:120502417	*BICDL1 (Body)*	−0.001	9.20E-01	0.044	2.93E-06*
cg23172057	chr13:113800351	*F10 (Body)*	0.010	1.55E-01	0.030	5.43E-08**
cg11042421	chr14:42881184	*—*	0.006	4.29E-02	0.015	2.71E-06*
cg14354244	chr14:51446038	*TRIM9 (Body)*	0.006	5.04E-01	0.034	3.66E-06*
cg03444659	chr14:94834215	*SERPINA2 (TSS1500)*	−0.002	7.14E-01	0.015	1.43E-06*
cg14792155	chr15:42289618	*PLA2G4E (Body)*	−0.007	7.50E-01	0.106	4.04E-06*
cg02187389	chr16:1247777	*CACNA1H (Body)*	0.000	9.97E-01	0.024	7.96E-07*
cg02234314	chr19:55986224	*ZNF628 (TSS1500)*	0.003	7.85E-01	0.059	2.08E-06*
cg07850633	chr20:15795880	*MACROD2 (Body)*	0.017	3.49E-01	−0.065	1.49E-06*
cg09772858	chr22:49549729	*—*	0.006	4.15E-01	0.030	2.52E-06*

CpG = cytosine-phosphate-guanine methylation site, FDR = False Discover Rate, NNNS = NICU Network Neurobehavioral Scale; For CpGs with no annotated genes, we annotated this with the nearest genes within 2500 bp of the CpG.

Based on observed differences in SES, maternal educational attainment, and maternal smoking during pregnancy among those with optimal or atypical NNNS profiles, as well as hypothesized potential confounding effects of batch (sample plate), immune cell proportions, race, and birth weight, we performed a sensitivity analyses with additional adjustment for these variables in our linear models. Additional adjustments for these potential confounders did not alter the observed associations between DNAm and atypical NNNS profiles (Supplemental Fig. 4).

### Functional and Phenotype Enrichment

We then used enrichment analyses to examine whether the genes annotated to NNNS-associated CpGs have a higher than expected proportion of genes that interact with each other, are involved in known biological pathways, or are linked to specific gene ontology terms. For this analysis, we utilized the top 250 CpGs that associated with the atypical NNNS profiles. We found that this gene-set was enriched for one neighborhood-based entity sets (NESTs) (FDR q-value = 0.031), centered on the *CRIM1* gene which has physical interactions with four genes, two of which were also in our gene-set: *ATXN7* and *MEGF6*. We also identified 54 pathway-based gene-sets (Supplemental Table 4) many of which may be relevant for neurodevelopment, including synaptic activity, neurotransmitters, and nerve growth factors (Table 4). Additionally, our gene-set was enriched for nine gene-ontology (G0) terms (Supplemental Table 5), including neuron projection (FDR q-value = 0.0371) and neuron part (FDR q-value = 0.0505). Multiple pathway and GO-term enrichments included *GRIK3*, *TRIM9*, and *PLA2G4E*, genes that were annotated to CpGs that yielded FDR-significant associations from our EWAS.

**Table 4:** Pathways involved in neurodevelopment and/or neuronal activity that were significantly (FDR < 0.10) over-represented among the genes annotated to the top 250 CpGs that associated with the Atypical NNNS profile.

Pathway ID	Pathway Description	Total Genes	NNNS-Associated Genes	P-val.	FDR Q-val.
path:hsa04724	Glutamatergic synapse - Homo sapiens (human)	114	*PLCB1*; *GRIK3*; *PRKCB*; *PLA2G4E*; *ADCY3*	0.0020	0.026
R-HSA-112314	Neurotransmitter receptors and postsynaptic signal transmission	152	*PLCB1*; *GRIK3*; *AP2A2*; *PRKCB*; *ADCY3*	0.0069	0.040
R-HSA-416993	Trafficking of GluR2-containing AMPA receptors	17	*PRKCB*; *AP2A2*	0.0077	0.040
R-HSA-112316	Neuronal System	367	*PLCB1*; *PRKCB*; *PTPRS*; *KCNK9*; *ADCY3*; *GRIK3*; *AP2A2*; *KCNA3*	0.0082	0.041
ngfpathway	nerve growth factor pathway (ngf)	18	*PLCG1*; *NGFR*	0.0086	0.043

NNNS = NICU Network Neurobehavioral Scale; FDR = False Discover Rate.

Since only one of our CpGs yielded an association with the NNNS profiles at a stricter threshold of statistical significance (5% FDR), we focused on this CpG for follow-up analyses examining whether the atypical NNNS profile exhibited differential methylation with each of the NNNS profiles. We regressed cg23172057 DNAm levels on a six level factor variable (using the atypical profile as the referent) while adjusting for sex, site, PMA, proportions of epithelial cells, and proportions of fibroblasts. The atypical NNNS profile exhibited significantly higher DNAm (p-values < 0.05) than each of the NNNS profiles, with the most substantial differential DNAm when comparing to Profile 3 (4.0% lower; p-value = 1.242E-07) and Profile 5 (3.4% lower; p-value = 9.76E-08) (Supplemental Fig. 5).

### CpG Annotation

Relatively few genes have been studied for their associations with neonatal neurobehavioral characteristics. However, it is plausible that the genes that are linked to cognition, neurobehavior, or neurodegeneration at other life stages may also be important in neurobehavioral function in very early life. We identified phenotypes or traits that have been associated with the genes annotated to the CpGs associated with NNNS profiles at a 10% FDR (Table 5). Importantly, Table 5 also presents 7 of the 11 genes that have been linked to neurobehavioral or neurodegenerative traits including autism, attention deficit hyperactivity disorder, cognitive impairment, depression, and psychosis.

**Table 5:** Genes annotated to our atypical NNNS-associated CpGs that have been linked to traits from the GWASdb.

Gene	N	Trait Types	Specific Cognitive, Neurobehavioral or Neurological Traits
*F10*	4	Hematopoietic	—
*EBF1*	34	Hematopoietic, Cardiovascular, Immune, Growth & Metabolism, Birth Outcomes, Neurobehavior, Cognition	Psychosocial stress measurement, cognitive impairment, cognitive decline measurement
*RGL1*	7	Neurobehavior, Cardiovascular, Environmental Exposures, Hematopoietic, Liver	Attention deficit hyperactivity disorder, conduct disorder, schizophrenia, response to antipsychotic drug, response to antidepressant
*CACNA1H*	1	Hematopoietic	—
*EBF3*	3	Cancer, Bone, Neurological, Sleep	Peripheral neuropathy
*SERPINA2*	6	Growth, Respiratory, Cardiovascular, Hematopoietic	—
*MACROD2*	31	Neurobehavior, Neurological, Liver, Growth & Metabolism, Hematopoietic, Bone, Birth Outcomes, Respiratory, Cardiovascular, Environmental Exposures, Bone, Immune	Autism, eating disorder, brain connectivity measurement, sporadic amyotrophic lateral sclerosis, prion disease, mood disorder
*ZFAT*	12	Growth & Metabolism, Cardiovascular, Cognitive, Environmental Exposures, Immune, Respiratory, Other	Self-reported educational attainment
*GRIK3*	6	Neurobehavior, Immune, Respiratory, Environmental Exposures	Unipolar depression, neuroticism measurement, depressive symptom
*TRIM9*	5	Growth & Metabolism, Neurobehavior, Immune, Cancer	Psychosis
*PLA2G4E*	1	Environmental Exposures	—

N = number of traits linked to that gene.

## Discussion

Our study focused on a comparison of neurobehavioral profiles with DNAm levels and epigenetic age. We used the NNNS summary scores to identify a group of infants with an optimal profile and a group with an atypical profile, which are similar to what has been observed previously by others and in our own research including preterm infants^21,24,29^. The infants in the atypical profile had the lowest scores for attention, self-regulation and quality of movement, average scores for lethargy, hypotonicity, nonoptimal reflexes and asymmetric reflexes, combined with the highest scores for arousal, excitability, hypertonicity and the most stress abstinence. This pattern of NNNS responses in our atypical profile was consistent with a latent profile identified by Liu *et al*. (2010) that was most predictive of non-optimal developmental outcomes in childhood^21^. Whereas the infants in our optimal profiles had the most positive responses across the NNNS and provided the greatest contrast to compare with the atypical profile. We found that very premature infants in the NOVI cohort with an optimal neurobehavioral profile had older epigenetic age than other very premature infants. We also found that age acceleration followed a stepwise trend in which infants with the optimal profile had the greatest age acceleration and infants with the atypical profile had the least age acceleration, though this finding was not statistically significant and may be driven by the differences in age at exam across the NNNS profile groups. Epigenetic age is an estimate of the state of underlying physiologic processes, as they relate to biological development and maintenance^32^, and has been studied in the context of health conditions that are linked to the aging process, including frailty^50^, physical capability^51,52^, cognitive fitness^51^, decreased cognitive function and neuropathologies in persons suffering from Alzheimer’s Disease^53^, and all-cause mortality^54^. In these studies, age acceleration is analogous to biological decline.

These relationships may differ in early life, however, when children are still undergoing substantial growth and development, and it is unclear whether accelerated aging would be expected to be associated with positive or negative developmental characteristics. In fact, if epigenetic aging captures, or is a surrogate for, the activity of developmental processes, epigenetic age acceleration throughout early development may be an indicator through which to track developmental “catch-up”. An epigenetic clock has also been developed to estimate epigenetic gestational age acceleration from cord blood DNAm^55^. Interestingly, gestational age acceleration has been associated with reduced infant respiratory morbidities^56^, which provides some evidence that older epigenetic age in infancy may correlate with positive developmental characteristics. In our analysis, we did not observe a significant relationships between age acceleration with the optimal or atypical NNNS profiles.

A handful of studies have examined the relationships between epigenetic age and development in children. For instance, epigenetic age positively correlates with measures of physical development such as fat mass, height, Tanner stages^33^, pubertal development, internalization and thought problems^34^, and increased cortisol^34,35^. One study observed interrelationships between age acceleration, cortisol production, and hippocampal volume, potentially linking hypothalamic-pituitary-adrenal (HPA) axis activity, neuroanatomy, and epigenetic aging^35^. This topic requires additional study and should ideally be investigated in a longitudinal manner, in which both epigenetic age and neurobehavioral assessments are tracked in parallel through early-life development.

Though the infants with the atypical NNNS profile did not have significantly different epigenetic age from the other NOVI infants, we did identify multiple differentially methylated CpGs throughout the genome that were associated with the group with atypical neurobehavioral responses. Our EWAS revealed 29 epigenetic loci that significantly associated (<10% FDR) with the atypical profile. The CpG with the smallest p-value was annotated to the (cg2317205) *F10* gene and was the only CpG that yielded an association at a stricter threshold for statistical significance (<5% FDR). We also found that the average DNAm at cg23172057 was significantly higher among the atypical NNNS profile even when comparing to each of the 5 other NNNS profiles individually. Thus, this CpG holds promise as a potential marker of problematic neurobehavioral responses in preterm infants. However, the gene that this CpG is annotated to (*F10*) has not been associated with behavioral or cognitive function. Instead, *F10* is involved in the blood coagulation cascade and primarily associated with hematologic disorders^57^. On the other hand, the other 28 CpGs only met a 10% FDR threshold of statistical significance, and likely do include some false positives, but a number of the genes annotated to these CpGs have been linked to cognition or educational attainment (*EBF1* & *ZFAT*), and neurobehavioral or neurological disorders in GWAS studies (*EBF1*, *RGL1*, *EBF3*, *MACROD2*, *GRIK3*, and *TRIM9*). We also found that the top 250 CpGs from this analysis were enriched for genes within pathways involving neurotransmitters and synaptic activity, as well as GO-terms related to neuron projection and structure. Of particular note, three genes annotated to the FDR-significant CpGs were within these neuronal-associated GO-terms and pathways: phospholipase A2 group IVE (*PLAG2G4E*), glutamate ionotropic receptor kainate type subunit 3 (*GRIK3*), and tripartite motif containing 9 (*TRIM9*). The CpG site that yielded the largest magnitude of association among our statistically significant hits, cg14792155, was within the body of the *PLA2G4E* gene. This gene encodes for a calcium-dependent N-acyltransferase; experimental mouse models have implicated that it likely plays a critical role in endocannabinoid signaling in the nervous system^58^ and thus differential regulation of this gene has implications for neurodevelopment and neurodegenerative disorders^59^. In human observational studies, placental CpGs within *PLA2G4E* have been observed to be differentially methylated in association with extremely preterm births^60^ and genetic variants within the *PLA2G4E* gene have been implicated as a potential risk factor for neurodevelopmental problems such as panic disorder^61^. The protein encoded by *GRIK3* is involved in presynaptic neurotransmission, and has been associated with developmental delay^62^, schizophrenia^63^, obsessive-compulsive disorder^64^, and depression^65^. The protein encoded by *TRIM9* regulates axon guidance and neural outgrowth^66,67^, while deletion of *TRIM9* has been associated with structural and functional abnormalities and impaired learning and memory in mice^68^. The CpG site that yielded the largest statistically significant inverse association, cg07850633, was within the body of the MACRO domain-containing protein 2 (*MACROD2*) gene; which has been associated with autism spectrum disorder (ASD)^69^, though other studies have yielded potentially contradictory evidence^70^, autism-like traits^71^and has been implicated in other neurological disorders^72,73^, and temporal lobe volume^74^. Variants in *EBF3*, which encode for the early B Cell Factor 3, may contribute to developmental delay and intellectual impairment^75^. Genetic variation in the Ral Guanine Nucleotide Dissociation Stimulator Like 1 (*RGL1*) gene, has been associated with attention^76^ and conduct problems among children with attention deficit hyperactivity disorder^77^. Additionally, genetic mutations within *CACNA1H*, which encodes for a subunit of a voltage gated calcium channel, lead to decreased calcium channel activity in neuronal cells, and have been linked to ASD^78^, to epilepsy^79^, and to amyotrophic lateral sclerosis^80^. Overall, these findings suggest that our NNNS-associated epigenetic variations occurred at numerous genomic regions with recognized roles in neurodevelopmental or neurodegenerative disorders. Interestingly, Sparrow *et al*. (2016) performed an EWAS of preterm birth using saliva samples to identify a number of genes that were differentially methylated in association with preterm birth that are also involved in neuronal function and/or neurobehavioral traits^81^. Their findings lead them to speculate that preterm-associated variations in DNAm may contribute to the neural and behavioral phenotypes that are linked to preterm birth. Thus, differential epigenetic regulation in babies that are born preterm may provide a link between preterm birth and poorer neurodevelopmental outcomes.

There were some limitations to this study. We used a false discovery rate of 10% to identify significantly differentially methylated CpG sites. Only one of our models yielded an FDR < 5%, and none of the models would survive Bonferroni adjustment. Thus, it is probable that some of identified epigenetic loci are false-positives. We encourage additional investigation of infant DNAm, epigenetic age, and neurobehavior to determine whether similar relationships can be observed in independent populations. There is also the possibility of residual confounding, though our findings were robust to adjustments for numerous potential confounders. We utilized buccal cells as a surrogate tissue to examine the relationships between neurobehavioral profiles and DNAm, as it is not possible to perform such examinations in the neuronal tissues. However, for studies of children, buccal cell collection leads to greater compliance^82^, and evidenced here in our 93% consent rate for parents who gave overall consent for NOVI. Recent evidence also suggests that buccal samples may be very appropriate for epigenetic analyses of neurodevelopmental outcomes, as they arise from the same germ cell layer as the brain and thus may share similar early epigenetic patterning and susceptibility^83–85^, and have demonstrated DNAm variability associated with later neurobehavioral outcomes.

We nevertheless remain cautious in the interpretation of these observations in terms of mechanism. These data were collected and analyzed cross-sectionally, so we cannot infer directionality of the observed relationships between NNNS profiles with DNAm or epigenetic age. It is notable, however, that the atypical profile observed by us and others in different populations^21,24,29^, has also been related to differential DNAm in other tissues^29^ and predicted developmental outcomes in childhood^21^. Thus, it is possible that the combination of epigenetic measures and NNNS profiles may lead to the early identification of which individual children are most at risk for adverse developmental outcome. Longitudinal studies of epigenomics and neurobehavioral outcomes are needed to establish whether epigenetic variations are detectable prior to the presentation of neurobehavioral impairments, and to examine whether and how these potential predictors vary throughout early life development.

## Conclusions

We found that among very preterm infants (<30 weeks PMA), those with an optimal neurobehavioral profile had slightly older epigenetic age, while infants with a poorly regulated neurobehavioral profile had differentially methylated CpGs at multiple genes linked to neural structure, function, or different neurobehavioral or neurodegenerative conditions. These relationships were detected using buccal cell DNAm, building upon the existing evidence that buccal cells may be a suitable surrogate tissue for studying neurobehavioral conditions in human observational studies. One CpG within the *F10* gene had the strongest association (<5% FDR) with the NNNS, while three other CpGs (<10% FDR) were within genes yielding multiple levels of evidence for plausible roles in neurobehavioral health, annotated to *PLA2G4E*, *TRIM9*, and *GRIK3*, all of which were among the significantly enriched GO-terms or neuronal pathways, and linked to neurobehavioral disorders. The combination of epigenomics and neurobehavior holds promise for a personalized medicine approach to the early detection of children most at risk for poor developmental outcome.

## Supplementary information

Supplemental Figures

Supplemental Tables

## Data Availability

The microarray data generated and/or analyzed in the current study are available in the NCBI GEO [Accession series GSE128821]. R codes used for the analyses presented in the paper are available upon request to the corresponding author.
