# Supplementary material for: Epigenome-wide Analysis Identifies Genes and Pathways Linked to Neurobehavioral Variation in Preterm Infants
Source: Sci Rep. 2019 Apr 19;9:6322. doi: 10.1038/s41598-019-42654-4 (PMC6474865; doi:10.1038/s41598-019-42654-4)

**Supplemental Materials**

**Title:** Epigenome-wide Analysis Identifies Genes and Pathways Linked to Neurobehavioral Variation in Preterm Infants

**Authors:** Todd M. Everson, Carmen J. Marsit, T. Michael O’Shea, Amber Burt, Karen Hermetz, Brian S. Carter, Jennifer Helderman, Julie A. Hofheimer, Elisabeth C. McGowan, Charles R. Neal, Steven L. Pastyrnak, Lynne M. Smith, Antoine Soliman, Sheri A. DellaGrotta, Lynne M. Dansereau, James F. Padbury, Barry M. Lester

**Supplemental Figure 1**: Distribution of the proportion of estimated epithelial cells within our buccal cell samples; for 95% of our samples epithelial cells made up greater than 95.7% of the cell types.


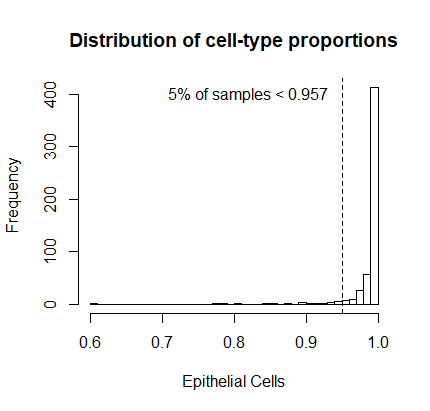


**Supplemental Figure 2**: Correlations between the proportions of immune cell types with the proportions of epithelial cell types; total immune cell proportions is equal to the sum of B-cells, NK cells, CD4+ T-cells, CD8+ T-cells, monocytes, neutrophils, and eosinophils.


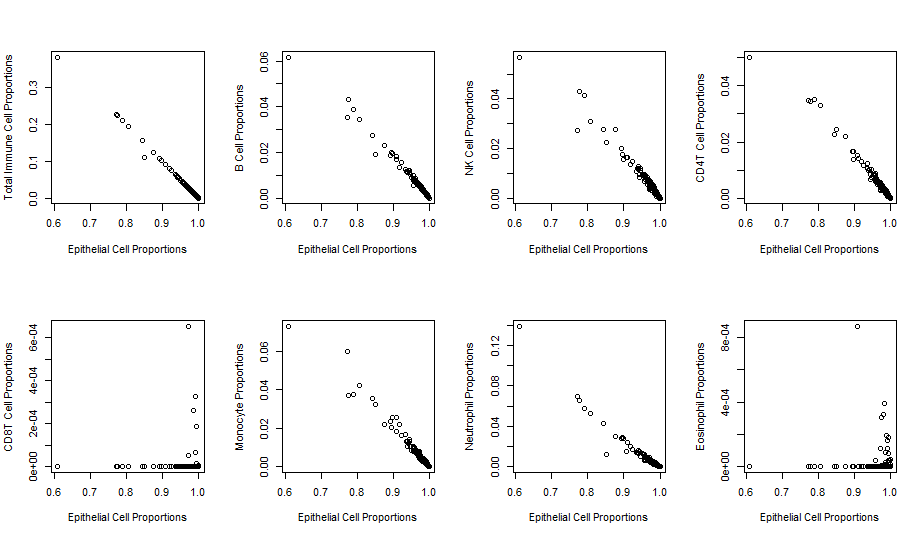


**Supplemental Figure 3:** Relationships between epigenetic age with gestational age, chronological age since birth, and postmenstrual age (different age variables on the x-axis represent days divided by 365 and can be interpreted as years).


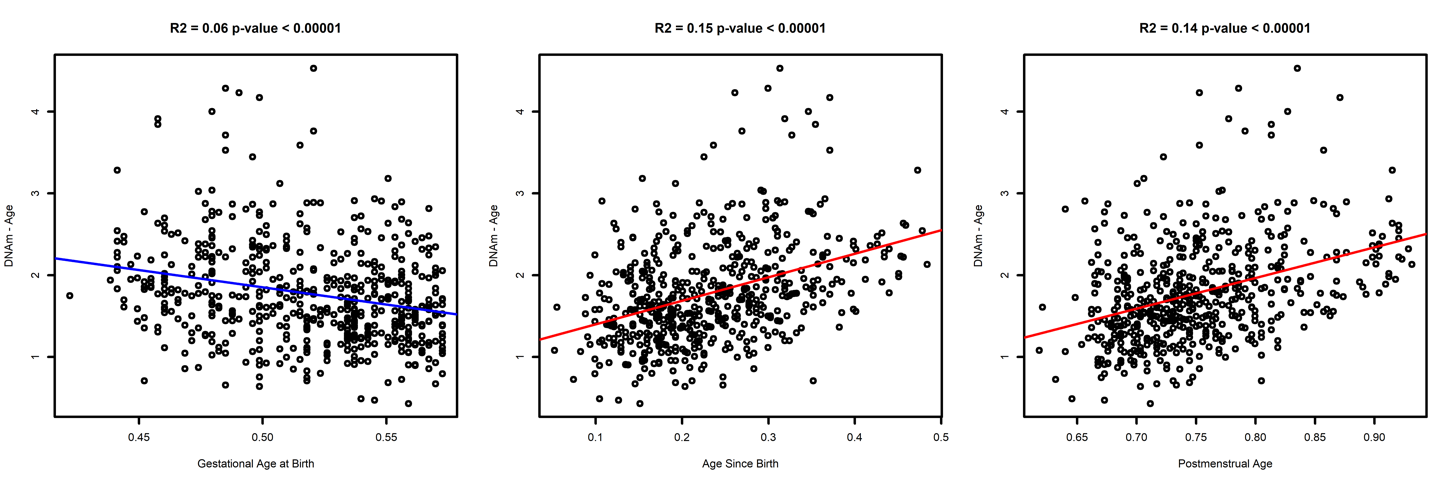


**Supplemental Figure 4:** Comparison of beta-coefficients (for the association between DNAm and the atypical NNNS profile) before and after adjustment for potential confounders including sample plate (A; 7-level factor), low SES (B; 2-level facor), low educational attainment (C; 2-level factor), maternal smoking during pregnancy (D; 2-level factor), additional cell type proportions (E; B-cells, natural killers, CD4+ T-cells, CD8+ T-cells, monocytes, neutrophils, eosinophils, and fibroblasts), race (F; 2-level factor), race (G; 5-level factor) and birth weight (H; grams) among the 30 CpGs that yielded the statistically significant (10% FDR) associations with the atypical or optimal NNNS profile.


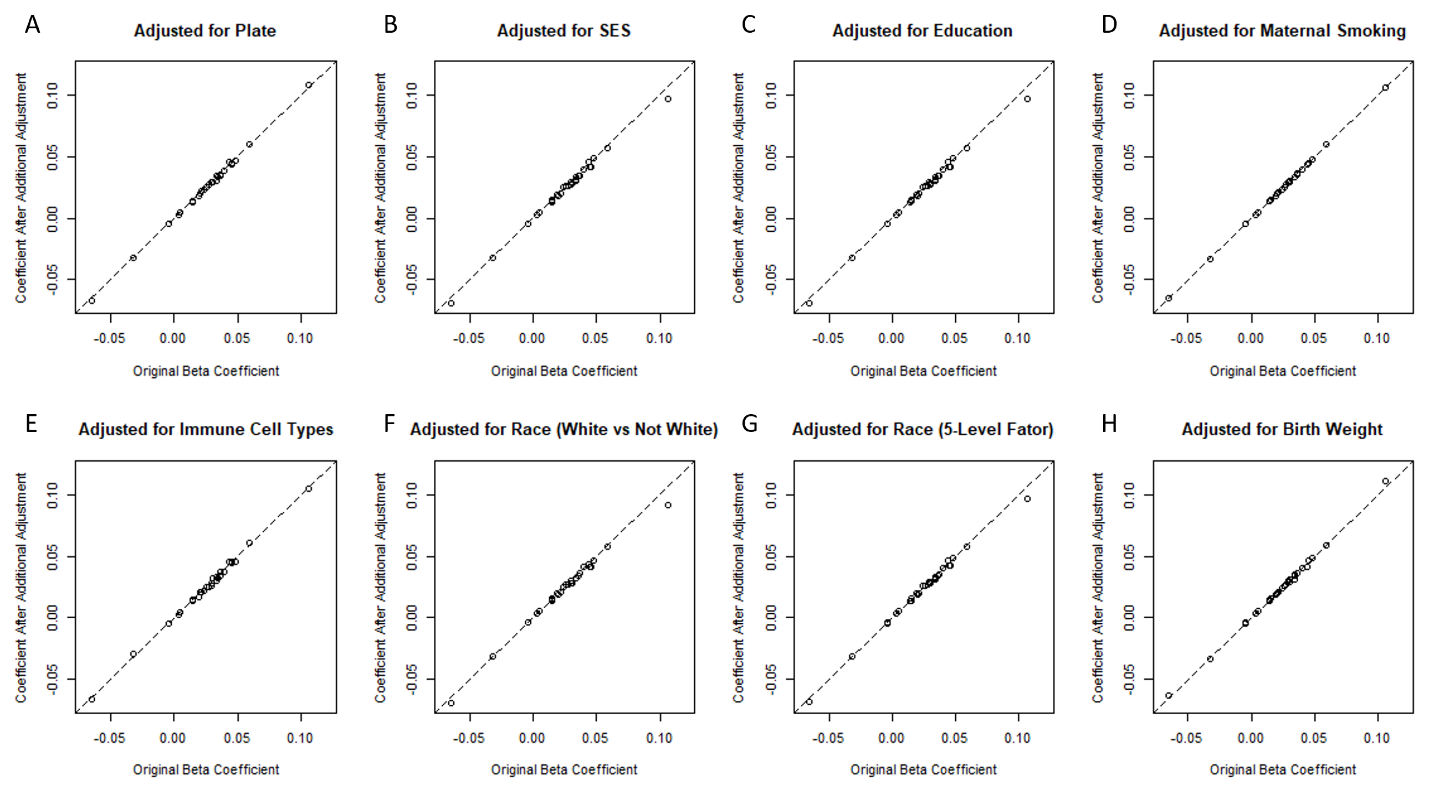


**Supplemental Figure 5:** Partial residual plot demonstrating the estimated mean (red lines) and 95% confidence intervals (shaded area) at cg23172057 among the different NNNS profiles, as well as the test (p-value) of differential methylation when comparing the atypical NNNS profile (reference group) to all other NNNS profiles while adjusting for sex, site, PMA, and epithelial cells.


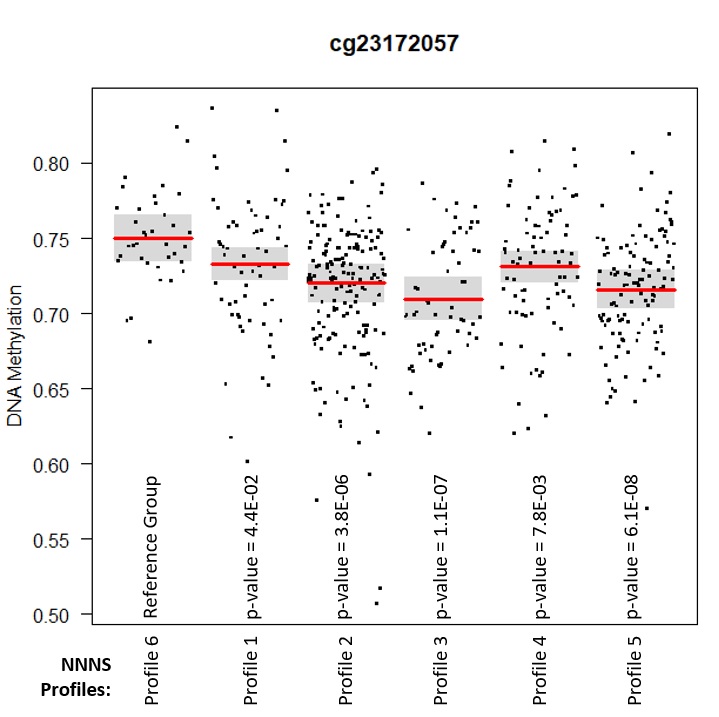

Supplement: Supplementary file 1 — Supplemental Figures [file 41598_2019_42654_MOESM1_ESM.docx]
